# Comparative Study of Free and Encapsulated Hypocrellin B on Photophysical-Chemical Properties, Cellular Uptake, Subcellular Distribution, and Phototoxicity

**DOI:** 10.3390/nano15120889

**Published:** 2025-06-09

**Authors:** Weiyan Kang, Feng Zhao, Jixing Cheng, Kaijie Feng, Liang Yan, Yue You, Jinxia Li, Jing Meng

**Affiliations:** 1 Department of Medicinal Chemistry, School of Pharmacy, Hebei Medical University, Shijiazhuang 050017, China; kangwy@ihep.ac.cn; 2 Lab for Biomedical Effects of Nanomaterials and Nanosafety, Institute of High Energy Physics, Chinese Academy of Sciences, Beijing 100049, China; zhaof@ihep.ac.cn (F.Z.); chengjx@ihep.ac.cn (J.C.); fengkj@ihep.ac.cn (K.F.); yanliang@ihep.ac.cn (L.Y.); youyue@ihep.ac.cn (Y.Y.)

**Keywords:** hypocrellin B, liposomes, PLGA nanoparticles, cellular uptake, subcellular localization, photodynamic therapy

## Abstract

The present study compared the free and encapsulated photosensitizer hypocrellin B (HB) in terms of photophysical-chemical properties, cellular uptake, subcellular distribution, and phototoxicity. The hydrophobic HB was encapsulated into liposomes (HB@Lipo) or poly (lactic-*co*-glycolic acid) nanoparticles (HB@PLGA). Encapsulation into nanocarriers exerted no obvious influence on the photophysical-chemical properties of HB, including UV-visible absorbance, fluorescence spectra, singlet oxygen (^1^O_2_) production capacity, and photostability. Free and encapsulated HB revealed some disparities in cellular uptake and subcellular localization patterns. In 2D-cultured B16 cells and tumor spheroids, free HB exhibited the fastest cellular uptake, while HB@PLGA had the lowest, as evidenced. Subcellular localization analysis first revealed a significant colocalization of free HB, HB@Lipo, and HB@PLGA within lipid droplets, with minimal colocalization in mitochondria and the endoplasmic reticulum. Unlike free HB and HB@Lipo, HB@PLGA exhibited strong lysosomal colocalization, indicating a unique intracellular trafficking pathway for PLGA-encapsulated HB. Upon laser irradiation, both free and encapsulated HB induced pronounced phototoxicity with substantial ROS production, confirming the robust PDT effect of HB. The photodynamic killing effect correlated with the intracellular HB content. These findings highlighted the impact of nanoformulation on HB’s cellular behavior and therapeutic performance.

## 1. Introduction

Photodynamic therapy (PDT) represents an innovative approach in oncology, leveraging light-mediated reactive oxygen species to exert remarkable therapeutic effects against tumors. Compared to conventional cancer treatments such as surgery, radiotherapy, and chemotherapy, PDT offers distinct advantages, including high therapeutic efficacy, absence of drug resistance, and minimal invasiveness. Although the low selectivity of photosensitizers and the limitations of light penetration depth, along with the hypoxic tumor microenvironment with heterogeneous vascular networks, might compromise PDT efficiency, especially for deeply seated tumors, developing next-generation photosensitizers with enhanced targeting capabilities and selectivity, coupled with the exploitation of nanotechnology and other therapeutic modalities, is expected to improve PDT efficiency [1]. Given its great advantages and promising potential for continuous improvement, PDT has become an increasingly attractive option in the clinical management of specific cancers [2,3,4]. With continued research development, it is anticipated that PDT will play an even more significant role in the future of cancer treatment.

In photodynamic therapy, photosensitizers are activated by light of a specific wavelength and produce reactive oxygen species (ROS), which can damage the diseased tissues. PDT efficiency depends largely on the photochemical, photobiological, and pharmacokinetic properties of photosensitizers. The ideal photosensitizer must possess a specific accumulation ability in tumor tissues, a relatively rapid clearance rate in normal tissues, strong absorption within the range of 600–850 nm, along with a high ROS yield. The absorption range of 600–850 nm is considered as the phototherapeutic window, within which endogenous chromophores (e.g., hemoglobin and water) exhibit minimal absorption and thus facilitate deeper and more efficient light penetration into the tissues. Since some photosensitizers possess broad absorption spectra, it is critical to recognize that the broad absorption of photosensitizers can be beneficial but also can lead to off-target activation or increased phototoxicity if parts of the spectrum extend beyond the therapeutic window. Therefore, the potential side effects arising from broad-spectrum photosensitizers should be highlighted when considering safety profiles. It is of great importance to achieve the optimization of photosensitizer properties and irradiation parameters to maximize therapeutic efficacy while minimizing unintended consequences.

Hematoporphyrin derivatives are widely used as the first generation of photosensitizers in PDT. However, subsequent studies revealed challenges in regulating their in vivo behaviors. This was attributed to their chemical instability and significant chemical heterogeneity [5]. Their metabolism in the body is relatively slow, and patients need to avoid light for a long time after treatment to prevent skin-photosensitive reactions [6]. Hypocrellin B (HB) is a naturally occurring photosensitizer isolated from the traditional Chinese medicine Hypocrella bambusea. In clinical practice, it also has certain applications in the treatment of skin diseases, such as psoriasis. Some studies have demonstrated that HB can inhibit tumor cell activity via photodynamic killing, although these investigations are still at the laboratory research stage. One study reported a significantly increased apoptosis and inhibited adhesion and migration in ovarian cancer cells after HB-activating PDT [7]. After light irradiation, HB significantly promoted nuclear fragmentation and induced apoptosis by inhibiting mitochondrial activity in A431 cells [8]. All these studies suggest that HB is expected to be used clinically in cancer treatment. 

Similar to the majority of hydrophobic photosensitizers that tend to aggregate readily in aqueous solutions [9], the clinical application of HB is also restricted by its poor water solubility. Such limitations pose obstacles to its efficient delivery and ultimately affect its therapeutic effectiveness. A suitable drug nanocarrier is expected to enhance its delivery efficiency while preserving its photodynamic activity.

Liposomes have been proven to be efficient and safe carriers for photosensitizers. Liposomes, the bilayer membrane structures composed of phospholipids and cholesterol, have demonstrated remarkable capabilities in encapsulating and delivering photosensitizers, thereby enhancing drug delivery efficiency [10,11,12]. Similarly, poly(lactic-*co*-glycolic acid) (PLGA), a biodegradable polymer material, has been approved by the US Food and Drug Administration (FDA) for use in drug delivery systems. PLGA has a hydrophobic core structure, tunable amphiphilicity, and excellent biocompatibility [13]. Thus, given the potential for clinical application, liposomes and PLGA are the most suitable drug delivery carriers.

As an indispensable element in photodynamic therapy, the subcellular distribution of photosensitizers plays a pivotal role in PDT efficacy. The conventional PDT normally functions on subcellular organelles, such as endoplasmic reticulum, mitochondria and lysosome, and consequently causes rapid cell death upon the light activation of photosensitizers. Different photosensitizers may differ in the subcellular distribution and induce PDT via different mechanisms. For example, endoplasmic reticulum-localizing photosensitizers such as hypericin promoted calreticulin (CRT) exposure through endoplasmic reticulum stress [14]. *m*-tetra(hydroxyphenyl) chlorin was observed to be localized in the endoplasmic reticulum (ER) and induced paraptosis upon laser illumination by ER perturbation [15]. A curcumin-based photosensitizer was reported to enhance PDT efficiency by simultaneously targeting lipid droplets and the endoplasmic reticulum [16]. A nucleus-targeting photosensitizer nanoassembly was reported to facilitate the release of damaged double-stranded DNA from tumor cells, thus leading to the activation of the Stimulator of Interferon Genes (STING) pathway [17]. Recently, a lipid droplet-targeting type I photosensitizer with enhanced superoxide anion production was reported to cause the accumulation of cellular lipid peroxidation upon irradiation [18]. In addition, the utilization of nanocarriers also impacts the internalization and subcellular distribution as compared with the free drugs [19,20]. For instance, compared to free doxorubicin, the application of PLGA-PEG micelles induced a slow and steady release of the drug and also demonstrated an enhanced cellular uptake [21]. As compared with the free photosensitizer Pheophorbide-α (Pheo), the delivery of Pheo by the poly(ethyleneglycol-*b*-*ϵ*-caprolactone) nanoparticles was predominately achieved via a direct collision of the nanoparticles with the membrane [22]. Therefore, in-depth investigations into the cellular uptake mechanisms and subcellular distribution patterns of photosensitizers with or without drug delivery systems are highly warranted. Such research is expected to offer novel insights into PDT improvements.

In the present study, liposomes and PLGA nanocarriers, two clinically approved nanoparticle-based delivery systems, were selected to encapsulate HB. First, the preparation parameters were systematically optimized to achieve high encapsulation efficiency and appropriate particle sizes. Then, cellular uptake of free and encapsulated HB was compared by quantifying the red fluorescence of HB both in the commonly-cultured B16 murine melanoma cells and the tumor spheroids, respectively. Then great emphasis was put on the subcellular distribution especially in the specific organelles including nuclei, lipid droplets, mitochondria, endoplasmic reticulum, and lysosomes. Finally, the photodynamic effects were explored and compared. Our study will provide a clue for the underlying mechanisms of action as well as the impact of nanocarriers on the intracellular localization and trafficking processes of drugs.

## 2. Materials and Methods

### 2.1. Preparation of Liposomal HB (HB@Lipo)

Liposomal HB was prepared using the thin-film hydration method followed by extrusion as described previously [23]. In brief, 12 mg of 1,2-dipalmitoyl-sn-glycero-3-phosphocholine (DPPC) (Shanghai Dibo Technology Co., Ltd., Shanghai, China), 3 mg of cholesterol (Beijing InnoChem Science & Technology Co., Ltd., Beijing, China), and 5 μL of HB (40 mM) dissolved in DMSO were accurately weighed, sonicated and dissolved in 5 mL of chloroform, and then the mixture was transferred to a rotating flask. Under reduced pressure, the mixture was subjected to rotary evaporation at low speed (1 rpm) for 2 h to remove the organic solvent, yielding a uniform thin lipid film. Next, 1 mL of PBS was added to the film, followed by ultrasonication for hydration. The liposome suspension was then successively extruded through 400 nm and 200 nm polycarbonate membranes using a liposome extruder (Avestin LiposoFast, Ottawa, ON, Canada) to obtain the homogeneous small liposomes with controlled particle size. The as-prepared liposomes were freeze-dried for 24 h and stored at −20 °C for the following study.

### 2.2. Preparation of HB-Loaded PLGA Nanoparticles (HB@PLGA)

HB@PLGA nanoparticles were prepared using the double emulsification method as described previously [24]. In order to establish an optimized method for preparing HB@PLGA, various formulation parameters were explored, including the type of aqueous phase, PLGA concentration, and the volume ratio of the organic phase/aqueous phase. Different combinations of these parameters were tested and the particle size and HB loading content of the obtained nanoparticles were characterized. Finally, the optimal formulation for HB@PLGA preparation was established as follows: 20 mg of PLGA (Beijing InnoChem Science & Technology Co., Ltd.) was fully dissolved in chloroform (135 μL) to form the oil phase and 20 μL of HB (40 mM) was added and completely dissolved via ultrasonication. The chloroformic solution was emulsified in 400 μL of a 4% PVA (Mw: 9–10 kDa, 80% hydrolyzed) (Sigma-Aldrich, Steinheim, Germany) solution using a sonicator in an ice-water bath (3 s on, 5 s off, for a total of 15 min). Then this emulsion was added into 10 mL of 1% PVA solution drop-wise and the mixture was again sonicated under the same conditions to form a compound emulsion. Afterwards, this emulsion was transferred to a round-bottom flask, and the organic solvent was removed by evaporation. The nanoparticles were collected by centrifugation and washed with distilled water three times. The HB@PLGA suspension was freeze-dried for 24 h and the lyophilized powder was obtained and stored at −20 °C for future studies.

### 2.3. Characterization

Size distribution and zeta potential of the prepared HB@Lipo or HB@PLGA were determined using dynamic light scattering (DLS) (NanoBrook Omni, Holtsville, NY, USA). The freeze-dried HB@Lipo or HB@PLGA were resuspended in purified deionized water and sonicated to obtain a suspension. Then the suspension was subjected to DLS analysis. The average particle size and zeta potential of HB@Lipo or HB@PLGA were represented as the average ± standard deviation of three independent measurements.

For the determination of the drug encapsulation efficiency, UV-visible spectrophotometry was used to quantify the content of HB loaded in the liposomes or PLGA nanoparticles. In brief, the freeze-dried HB@Lipo or HB@PLGA nanoparticles were dissolved in DMSO and then the supernatant was separated by centrifugation. HB concentration in the supernatant was measured spectrophotometrically at 470 nm using a calibration curve plotted over a series of concentrations (0, 6.25, 12.5, 25, 50, and 100 μM) of pure HB in DMSO. Empty liposomes or PLGA nanoparticles were used as controls. According to the standard curve, the HB mass in the supernatant after the breakdown of nanoparticles by DMSO was calculated. Thus, the mass of HB loaded in the nanoparticles was obtained. Drug encapsulation efficiency (EE, %) was calculated using the formula:

EE (%) = (mass of HB loaded in nanoparticles/mass of HB processed) × 100,

where the mass of HB processed represents the total mass of HB initially added during nanoparticle preparation.

The UV-visible and fluorescence spectra of HB, HB@Lipo, and HB@PLGA were recorded using a UV-visible spectrophotometry and a fluorescence spectrometer, respectively. The singlet oxygen (^1^O_2_) production capacity of HB, HB@Lipo, and HB@PLGA was quantitatively assessed using UV spectrophotometry. Briefly, 1,3-diphenylisobenzofuran (DPBF), a ^1^O_2_ quencher, was dissolved in DMSO to prepare a 27 μM working solution. Subsequently, HB, HB@Lipo, or HB@PLGA was added to the DPBF solution to achieve a final HB concentration of 1 μM. The mixtures were irradiated (0.5 W/cm^2^, 10 s) and the absorbance at 415 nm was measured to quantify DPBF degradation, which reflects ^1^O_2_ generation.

The photochemical stability of HB, HB@Lipo, and HB@PLGA was evaluated by monitoring their UV-visible absorbance profiles under light exposure. Samples with an equivalent HB concentration of 10 μM, were irradiated (0.5 W/cm^2^, 5 min) and the absorbance changes at the respective maximum absorption wavelengths were recorded at defined intervals to assess the photodegradation kinetics.

### 2.4. Cellular Uptake of HB, HB@Lipo and HB@PLGA Analyzed Using Flow Cytometry

B16 cells were maintained in RPMI 1640 medium supplemented with 10% fetal bovine serum, 100 μg/mL penicillin, and 100 μg/mL streptomycin at 37 °C in a humidified atmosphere containing 5% CO_2_. Cellular uptake of HB, HB@Lipo, and HB@PLGA was examined using flow cytometry based on the red fluorescence of HB. The exponentially growing B16 cells were seeded into 6-well plates at a density of 2 × 10^5^ cells per well and incubated overnight. Then the cells were treated with HB, HB@PLGA, or HB@Lipo with an equivalent HB concentration of 2 μM for different time intervals (0, 1, 3, 6, 12, and 24 h). At each time point, cells were washed with PBS three times to remove the uninternalized HB. Cells were collected by centrifugation and the red fluorescence of HB in each sample was detected using flow cytometry.

### 2.5. Subcellular Localization of HB, HB@Lipo, and HB@PLGA Observed Using Confocal Laser Scanning Microscopy

Confocal laser scanning microscopy (CLSM) was used to observe the subcellular localizations of HB, HB@Lipo, and HB@PLGA after their internalization into cells. In brief, the well-growing cells were seeded into the confocal dishes (glass-bottom, Biosharp Life Sciences, Hefei, China) and cultured overnight. Then the cells were treated with HB, HB@Lipo, or HB@PLGA with an equivalent HB concentration of 2 μM for 4 h. After the removal of the medium, cells were washed with cold PBS three times and then stained with the corresponding fluorescent dyes in the dark for 15 min: Hochest 33342 (1 μM, Thermo Fisher Scientific, Waltham, MA, USA) for nuclei staining, BODIPY 505/515 (1 μM, Thermo Fisher Scientific) for lipid droplet labeling, Mito-Tracker Green (1 μM, Melone Pharmaceutical Co., Ltd., Dalian, China) for mitochondrion labeling, ER-tracker Green (0.5 μM, Biosharp Life Sciences) for endoplasmic reticulum labeling and Lyso-tracker Green (0.05 μM, Beyotime Biotechnology, Haimen, China) for lysosome labelling. After staining, the cells were washed three times with PBS and loaded onto a confocal scanning microscope. The subcellular localization of the photosensitizers was observed and the representative images were captured. The colocalization coefficient (Pearson correlation coefficient) in each group was analyzed using ImageJ software 1.8.0_345 (64-bit).

### 2.6. Culture of Tumor Spheroids

Tumor spheroids were cultured according to the procedures reported in the previous literature [25]. In brief, ultra-low attachment 96-well plates were utilized to maintain cell suspension and promote spheroid formation. B16 cells in the logarithmic growth phase were cultured in the ultra-low attachment plates at a density of 400 cells per well. Half of the medium was replaced with fresh medium every two days. The growth of tumor cell spheroids was continuously monitored and the diameter was measured under a microscope. The spheroids reaching a diameter size of 500 μm were selected for the following experiments.

### 2.7. Distribution of HB, HB@Lipo or HB@PLGA in Tumor Spheroids

When the diameter of the cultured spheroids reached 500 μm approximately, they were treated with HB, HB@Lipo, or HB@PLGA at an equivalent HB concentration of 2 μM. After incubation for 4, 12, and 24 h, the tumor spheroids were transferred to a confocal dish (glass-bottom, Biosharp Life Sciences) using a pipette. After PBS wash, the cellular uptake and distribution of HB, HB@Lipo, and HB@PLGA within tumor spheroids were observed and recorded using confocal laser scanning microscopy.

### 2.8. MTT Cell Viability Assay

MTT assay is a commonly used technique to evaluate cell viability and growth. MTT, (3-(4,5-dimethylthiazol-2)-2,5-diphenyltetrazole bromide), a yellow water-soluble dye, can be reduced by succinate dehydrogenase in live cell mitochondria to form purple insoluble methylene blue (Formazan). The amount of produced methylene blue indirectly reflects the number of living cells. Within a certain range of the number of cells, the amount of MTT crystal formation is directly proportional to the number of living cells [26].

The effects of HB, HB@Lipo, and HB@PLGA on B16 cell viability with or without laser illumination were evaluated using MTT assay. Cells were seeded into 96-well plates at a density of 5000 cells per well and cultured for 24 h. Then the cells were incubated with serum-free medium containing HB, HB@Lipo, or HB@PLGA (0, 0.25, 0.5, 1, 2, and 4 μM), respectively, for 6 h followed by laser illumination at a power density of 0.5 W/cm^2^ for 2 min. After an additional 18 incubation, the medium was discarded and a 10% MTT working solution was added to each well. Afterwards, cells were incubated for 3 h at 37 °C in the dark to allow formazan crystal formation. The MTT working solution was carefully removed and 100 μL of DMSO was added to dissolve the formazan crystals. After shaking, the absorbance was measured at 490 nm using a microplate reader (Thermo Scientific, Waltham, MA, USA). The well with DMSO added served as a blank group. In the dark toxicity evaluation, the cells without HB treatment served as the control. For the phototoxicity evaluation, cells treated with light alone served as the control. Cell viability was calculated according to the following formula:

Cell viability (%) = 100 × (OD_experimental_ − OD_blank_)**/**(OD_control_ − OD_blank_)

### 2.9. ROS Detection Using DCFH-DA Staining

Intracellular ROS was detected using dichlorodihydrofluorescein diacetate (DCFH-DA) assay kit. DCFH-DA, cell permeable fluorescent probe, can be taken up by cells where the cellular esterase cleaves off the acetyl groups. The resulting DCFH can be oxidized into DCF by intracellular ROS. DCF emits green fluorescence at an excitation wavelength of 485 nm and an emission wavelength of 530 nm. The fluorescence intensity indirectly reflects the ROS level [27]. In brief, B16 cells were seeded in 35 mm dishes and cultured overnight. Then the cells were incubated HB, HB@Lipo or HB@PLGA at an equivalent HB concentration of 1 μM for 6 h, followed by 2 min of laser irradiation. Then cells were cultured for an additional 2 h to allow ROS generation. After PBS wash, the cells were stained with DCFH-DA (10 μM) for 20 min. After rinse with PBS, cells were loaded onto a confocal laser scanning microscope (Nikon A1, Tokyo, Japan). The representative images were captured.

### 2.10. Statistical Analysis

All the experiments in the current study were independently repeated at least 3 times with similar results. The relative percentage and values were presented as the mean standard deviation (SD) of six parallel samples. Statistical analysis was performed by two-tailed Student’s *t*-test for unpaired data, with *p* < 0.1 considered statistically significant

## 3. Results

### 3.1. Preparation and Characterization of HB@Lipo

**Figure 1:**
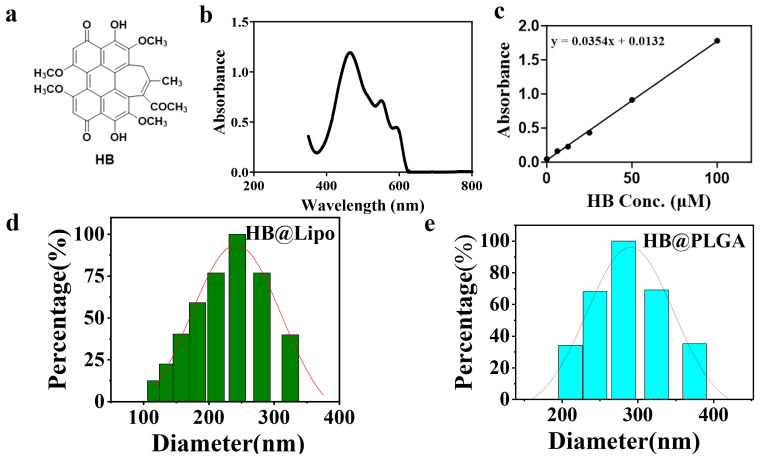
(**a**) Chemical structure of HB; (**b**) UV-Visible absorption spectrum of HB in DMSO; (**c**) The standard curve showing the linear relationship between HB concentrations and absorbance values at 470 nm; (**d**) Particle size distribution of HB@Lipo analyzed using DLS; (**e**) Particle size distribution of HB@PLGA analyzed using DLS.

The chemical structure of HB is shown in Figure 1a. In laboratory research, the thin-film hydration method followed by extrusion through polycarbonate membranes is a well-established technique for preparing homogeneous liposomes. In the current study, HB@Lipo was prepared as described in “Materials and Methods” section and the key parameters were demonstrated in Table 1.

To initiate the preparation of HB@Lipo, 12 mg of 1,2-dipalmitoyl-sn-glycero-3-phosphocholine (DPPC), 3 mg of cholesterol, and 5 μL of 40 mM HB (prepared in DMSO) were completely dissolved in 5 mL of chloroform. Subsequently, the resulting mixture was carefully evaporated under reduced pressure for the removal of chloroform. This process led to the formation of a thin lipid film uniformly coating the inner surface of the round-bottom flask. Then the lipid film was hydrated with PBS. Sonication was applied during the hydration process to ensure proper dispersion of the lipid film.

Following hydration, the mixture was extruded sequentially through polycarbonate membranes of 400 nm and 200 nm. This extrusion step helped to reduce the particle size and achieve more uniform HB-loaded liposomes (HB@Lipo).

To accurately quantify the amount of encapsulated HB, the absorption wavelength of HB was first determined using a UV spectrophotometer. The analysis revealed that HB had three characteristic absorbance bands at 470, 552, and 592 nm, with the maximum absorption peak at 470 nm (Figure 1b). Subsequently, the linear relation between HB concentrations and absorption values at 470 nm was explored. 40 mM stock solution of HB was serially diluted to the concentrations of 100, 50, 25, 12.5, and 6.25 μM. The absorbance of these solutions at 470 nm was measured using a UV spectrophotometer with DMSO as the blank control. Based on HB concentrations and the corresponding absorbance values, a standard curve was obtained, which yielded the equation (y = 0.0354x + 0.0132), with a correlation coefficient to be measured as 0.9987 (R^2^ = 0.9987) (Figure 1c). This result indicated that the absorbance values determined by spectrophotometry were linearly correlated with HB concentrations within the range of 0–100 μM. According to the standard curve, the encapsulation efficiency of the as-prepared HB@Lipo was calculated to be 80%.

Afterwards, the size distribution and zeta potential of the prepared HB@Lipo were characterized using dynamic light scattering (DLS) analysis. The results showed that HB@Lipo had an average hydrated particle size of 243.48 ± 5.23 nm with a zeta potential of −28.31 ± 0.68 mV (Figure 1d).

### 3.2. Preparation and Optimization of HB@PLGA

To prepare the HB-loaded PLGA nanoparticles, a systematic exploration of multiple parameters is essential to establish an optimized protocol, including the type and concentrations of aqueous phase (such as distilled water, polyvinyl alcohol), the concentration of PLGA, the volume ratio of the organic phase to the aqueous phase and the method of organic solvent removal [28]. Among these influencing factors, the concentrations of PLGA and aqueous phase are considered the most crucial variables. In the current study, polyvinyl alcohol (PVA) was selected for the aqueous phase and the concentration of PVA for the external aqueous phase was fixed at 1%. The concentration of HB was 40 mM. The organic solvent was removed by rotary evaporation for 1 h. By adjusting the proper concentrations for PLGA and PVA in the internal aqueous phase, the optimal synthesis conditions were explored. The particle size and encapsulation efficiency of the obtained nanoparticles were characterized to evaluate the formulated protocol. Generally, larger nanoparticles (>300 nm) show a reduced efficiency in cellular internalization, partially due to the prolonged membrane wrapping time and the requirement for specialized protein-mediated processes (e.g., clathrin or caveolin-dependent endocytosis). These energy-consuming mechanisms not only slow down the uptake kinetics but also enhance the clearance by the reticuloendothelial system. Therefore, in our study, we aimed to control the average particle size of the prepared nanoparticles within 300 nm while simultaneously optimizing the encapsulation efficiency [29,30]. Meanwhile, the zeta potential was also detected for a more comprehensive evaluation of the prepared nanoparticles. Since most cellular membranes are negatively charged, zeta potential can affect a nanoparticle’s tendency to permeate cell membranes. While anionic nanoparticles exhibit prolonged intracellular retention, their cationic counterparts show higher biotoxicity associated with cell membrane disruption [31]. Given the used PLGA is negatively charged, the prepared HB-loaded PLGA nanoparticles are inferred to carry a negative charge. The nanoparticles with a larger zeta potential (in absolute value) indicate a relatively strong electrostatic repulsion between particles, which is advantageous for improving the stability of the nanoparticle system [32]. Therefore, the zeta potential of the prepared HB-loaded PLGA nanoparticles was characterized in the present study.

In the preparation of PLGA nanoparticles, first, PLGA concentration was set at 10%, while PVA concentrations in the internal aqueous phase were varied within the range from 1% to 5%. The results are illustrated in Table 2. It was found that HB could not be effectively encapsulated within PLGA nanoparticles when PVA concentration in the internal aqueous phase was 3% or lower. The 0% encapsulation efficiency suggested that the empty PLGA nanoparticles were obtained when the PVA concentration for the internal aqueous phase was lower than 3%. This phenomenon may be attributed to insufficient viscosity caused by the low PVA concentration. Such low viscosity hindered the formation of well-structured microspheres. Additionally, the lack of a substantial osmotic gradient between the internal and external aqueous phases likely led to the extensive diffusion of the drug into the external aqueous phase, resulting in the failure of drug encapsulation.

Then, the PVA concentration for the internal aqueous phase was increased to 4% or 5%, and a remarkable improvement in the encapsulation efficiency of PLGA nanoparticles was observed. However, this increase in encapsulation efficiency was accompanied by a significant increase in the particle size as demonstrated by the comparison of the 4% PVA and 5%PVA group. This increased particle size might be attributed to the rise in the viscosity of emulsion resulting from the increased PVA concentration. This made it considerably more difficult to break down compound emulsion droplets into smaller ones.

Subsequently, the influence of PLGA concentration on the properties of nanoparticles was investigated with PVA concentration in the internal aqueous phase set at 4% or 5%. The concentration of PLGA was tested for 10%, 15%, and 20%. As illustrated in Table 3, although a higher PLGA concentration of up to 15–20% substantially enhanced the encapsulation efficiency, it also led to a significant rise in the particle size and Dispersity (*Đ*). This suggested that the elevated PLGA concentration was not favorable for the nanoparticle dispersion. Collectively, a combination of 10% PLGA and 4% PVA in the internal aqueous phase was determined to be the most optimal formulation. This combination effectively balanced both the encapsulation efficiency and the average diameter of the nanoparticles, providing a favorable compromise between these two crucial parameters.

Finally, the optimal formulation parameters were successfully determined and the physicochemical properties of the prepared HB@PLGA were summarized in Table 4. The optimal parameters were established as follows: 10% PLGA, 4% PVA in the internal aqueous phase, 1% PVA in the external aqueous phase, 20 μL of 40 mM HB in DMSO, and a volume ratio of HB solution to the oil phase of 1:18.7. The ultrasonic power was set to 40%, with sonication times of 15 min for both the primary and secondary emulsions. The volume of the external aqueous phase was maintained at 7.5 mL. These obtained HB@PLGA nanoparticles had an average particle size of 281.17 ± 1.26 nm (Figure 1e). The zeta potential was determined to be −48.37 ± 4.86 mV. The encapsulation efficiency of HB in the PLGA nanoparticles was determined to be 73%.

### 3.3. Photophysical-Chemical Properties of HB,HB@Lipo, and HB@PLGA

**Figure 2:**
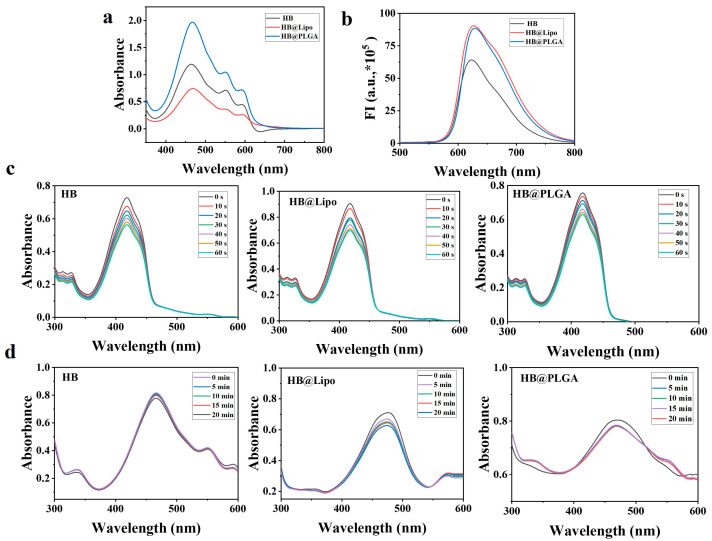
Absorption spectra (**a**) and fluorescence spectra (**b**) of HB, HB@Lipo, and HB@PLGA dissolved in DMSO; (**c**) Single linear oxygen (^1^O_2_) determination of HB, HB@Lipo, and HB@PLGA in DMSO; (**d**) Photodegradation determination of HB, HB@Lipo, and HB@PLGA in DMSO.

Figure 2a shows the absorption spectra of HB, HB@Lipo, and HB@PLGA dissolved in DMSO, which exhibited a similar pattern with maximum absorption at 470 nm. When excited at 470 nm, HB, HB@Lipo, and HB@PLGA were all observed to have fluorescence emission peaks at 620 nm in DMSO (Figure 2b).

The singlet oxygen (^1^O_2_) production capabilities of HB, HB@Lipo, and HB@PLGA in DMSO were evaluated using DPBF as a ^1^O_2_ quencher. Specifically, HB, HB@Lipo, or HB@PLGA was added into the DPBF solution followed by irradiation (0.5 W/cm^2^, 10 s). The absorbance at 415 nm was measured to quantify DPBF degradation, which reflected ^1^O_2_ generation. A decrease in the DBPF absorption band at 415 nm was observed in each sample, suggesting that HB, HB@Lipo, and HB@PLGA all triggered ^1^O_2_ generation. Time-dependent spectral analysis revealed that both HB@Lipo and HB@PLGA maintained high ^1^O_2_ generation rates comparable to free HB, demonstrating that encapsulation within nanocarriers did not compromise the singlet oxygen (^1^O_2_) production capability of HB. The sustained ^1^O_2_ production capacity of both free and encapsulated HB confirms their potential for photodynamic applications (Figure 2c).

The photochemical stability of HB, HB@Lipo, and HB@PLGA in DMSO was evaluated by monitoring their UV-visible absorbance profiles under light exposure. Samples of HB, HB@Lipo, and HB@PLGA with an equivalent HB concentration of 10 μM, were irradiated at 0.5 W/cm^2^ for 5 min and the absorbance changes at 470 nm were recorded at 5-min intervals to assess the photodegradation kinetics. The results from photodegradation studies showed that HB, HB@Lipo, and HB@PLGA exhibited average stability in DMSO.

### 3.4. Cellular Uptake and Subcellular Localization of HB, HB@Lipo and HB@PLGA

The intracellular transport and distribution of photosensitizers are critical factors influencing PDT efficiency. In this study, we investigated the intracellular localization and trafficking processes of free and encapsulated HB.

**Figure 3:**
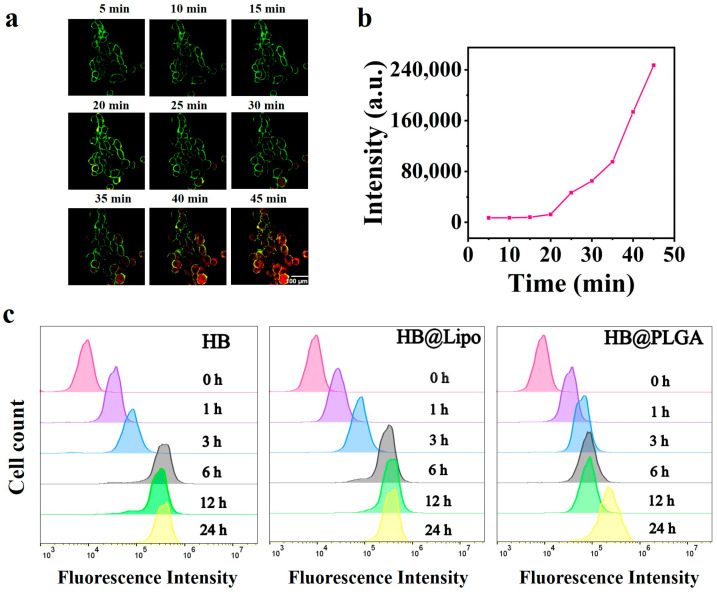
(**a**) Cellular uptake kinetics of HB in live B16 cells observed using CLSM. Cell membrane was stained with CellMask™ stain (green); (**b**) Normalized fluorescence intensity of intracellular HB at specified time points. (**c**) Cellular uptake of HB, HB@Lipo, and HB@PLGA analyzed using flow cytometry.

First, we exploited the red fluorescent properties of HB [33] to assess cellular uptake and intracellular distribution patterns. The uptake kinetics of HB within living B16 cells was monitored using CLSM. The cell membrane was stained with CellMask™ plasma membrane stain and can be visualized in the green channel (488 nm). The observation periods started from the beginning of HB added into cells and persisted for 45 min. Rapid incorporation of red fluorescent HB into tumor cells was detectable after 25 min of incubation and then the fluorescence intensity of intracellular HB increased rapidly, indicating a time-dependent HB internalization (Figure 3a,b).

Subsequently, a comparative analysis of the cellular uptake within 24 h was conducted among HB, HB@Lipo, and HB@PLGA. B16 cells were incubated with HB, HB@Lipo, or HB@PLGA with an equivalent HB concentration of 2 μM. The cellular uptake of HB, HB@Lipo, and HB@PLGA was detected using flow cytometry based on the red fluorescence of HB. The results clearly revealed that cellular uptake of HB, HB@Lipo, and HB@PLGA began as early as 1 h after being into cells and exhibited a time-dependent increase. Notably, cellular uptake of both free HB and HB@Lipo completed cellular uptake at 6 h, with the highest intracellular red fluorescence reached at this time point. Whereas, the cellular uptake of HB@PLGA was much slower and continued to increase over time until reaching its maximum at 24 h (Figure 3c). This curve in uptake kinetic suggested that the intracellular delivery and retention of HB was influenced by nanoformulations. Nevertheless, all the formulations exhibited comparable overall cellular uptake levels at 24 h.

After entering the cells, the subcellular localization of HB, HB@Lipo, and HB@PLGA was observed using CLSM. Special attention was paid to the distribution of HB, HB@Lipo, and HB@PLGA in the specific subcellular organelles including nuclei, lipid droplets, mitochondria, endoplasmic reticulum, and lysosomes. In all of the subsequent staining procedures, nuclei were stained with Hoechst 33342. After cells were incubated with HB, HB@Lipo, or HB@PLGA, no red fluorescence of HB was observed within the nuclei, indicating that no HB was taken up by the nuclei during the incubation periods.

**Figure 4:**
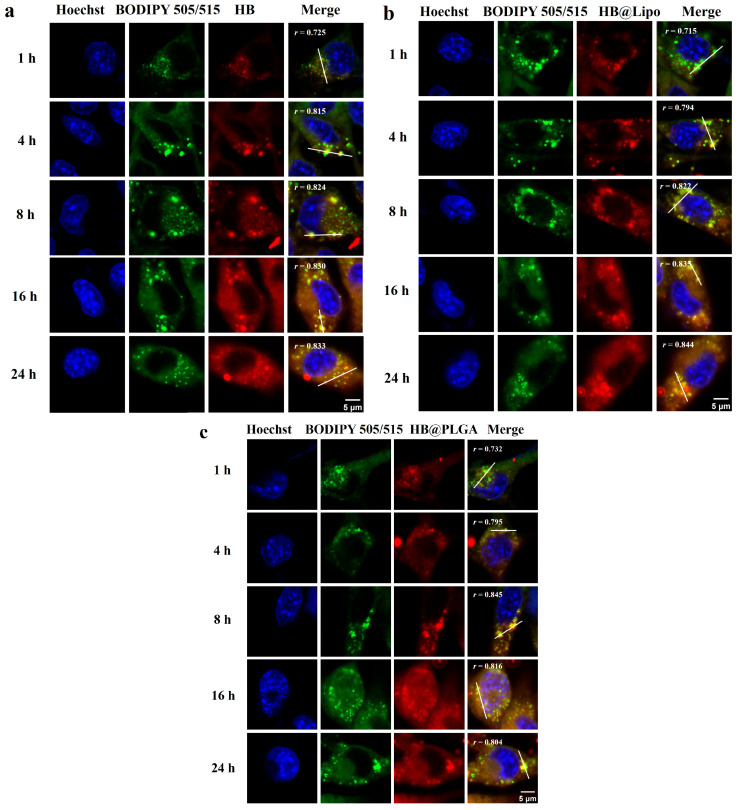
Colocalization of HB (**a**), HB@Lipo (**b**), and HB@PLGA (**c**) within lipid droplets in B16 cells. Scale bars: 5 μm. Pearson correlation coefficient (*r*) in each group was analyzed using ImageJ software.

Given the lipophilic characteristics of both HB and HB@Lipo, along with the structural similarity between liposomes and cell membranes, it is speculated that the cellular uptakes of HB and HB@Lipo are closely associated with their interactions with cell membranes and intracellular lipid droplets. Lipid droplets, once considered as simple fat storage sites, have been reported to play a critical role in cellular metabolism as dynamic organelles in recent years [34]. In the current study, lipid droplets within cells were labeled with BODIPY 505/515 and visualized as green granular fluorescence. The potential colocalization of HB, HB@Lipo, or HB@PLGA within lipid droplets was monitored at 1, 4, 8, 16, and 24 h. The results from CLSM observation demonstrated that there was a significant colocalization of red fluorescence and green fluorescence in the cells that were incubated with either HB or HB@Lipo. Pearson correlation coefficient, used for quantifying the colocalization correlation (*r*) [35] at all the time points, ranged from 0.7 to 0.9 (Figure 4a,b), suggesting a high and continuous colocalization of HB or HB@Lipo within lipid droplets. Moreover, HB@PLGA also exhibited a similar colocalization within lipid droplets (Figure 4c). These findings suggest that lipid droplets are the essential sites for the retention of HB and its nanoparticles after they enter the cells.

**Figure 5:**
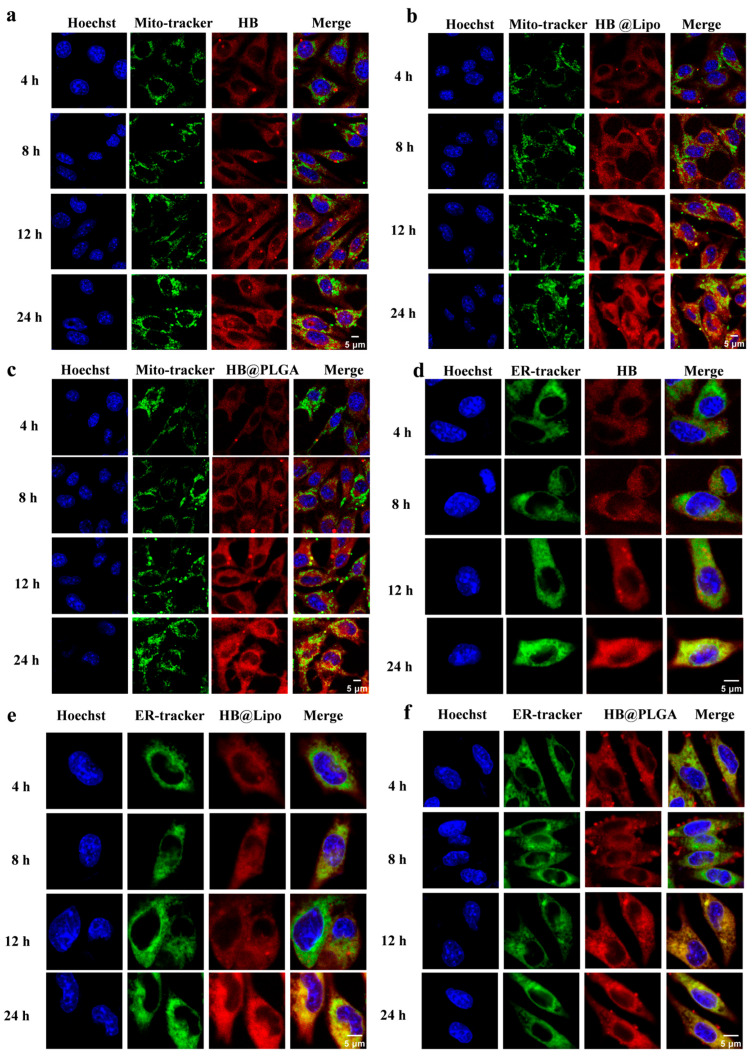
Representative CLSM images demonstrating the distribution of HB, HB@Lipo, and HB@PLGA in the mitochondria and the endoplasmic reticulum of B16 cells at different time points. (**a**,**d**) HB, (**b**,**e**) HB@Lipo, (**c**,**f**) HB@PLGA. Scale bar: 5 μm.

Mitochondria are the critical regulators of numerous biological pathways, including apoptosis induction, ROS generation, and mitochondrial DNA regulation. When photosensitizers specifically localize within mitochondria, the generated ROS upon light activation can immediately and efficiently initiate the mitochondrial apoptosis pathway, which significantly enhances PDT efficacy [36]. Cells were incubated with HB, HB@Lipo, or HB@PLGA for different time intervals and their localizations in Mito-Tracker Green-labeled mitochondria were examined carefully. As demonstrated in Figure 5a, in the cells incubated with HB, although the red signal intensity of intracellular HB increased over time, no obvious overlap with the green mitochondrial signals could be observed. Similarly, HB@Lipo or HB@PLGA, after cellular uptake, also did not retain in mitochondria (Figure 5b,c). These findings indicated that HB, HB@Lipo, or HB@PLGA did not target mitochondria after entering the cells.

Endoplasmic reticulum (ER) is a crucial membrane system within the cytoplasm, connecting the cell membrane and the nuclear membrane, and facilitating intracellular material transport [37]. More importantly, ER plays an important role in protein homeostasis. The foreign material that enters the cells may disrupt ER homeostasis and provoke a state of ER stress that would influence the diverse functions of cells [38]. In this study, the possible localization of HB and its nanoparticles in the endoplasmic reticulum was examined. To visualize the endoplasmic reticulum, the endoplasmic reticulum of B16 cells was labeled with ER-Tracker Green. Although the cellular uptake of HB increased over time, the degree of overlap between HB and the ER remained relatively weak, suggesting that ER was not the target site for HB (Figure 5d). Similarly, in the cells incubated with HB@Lipo (Figure 5e) or HB@PLGA (Figure 5f), no obvious colocalization was observed at 4, 8 and 12 h. A slight overlap was observed between the red HB signal and the green ER signal at 24 h. This might be due to the passive diffusion of HB into the ER when a greater amount of HB, HB@Lipo, or HB@PLGA was internalized into cells as the incubation time extended. It demonstrated that HB, HB@Lipo, or HB@PLGA probably did not specifically target the endoplasmic reticulum.

**Figure 6:**
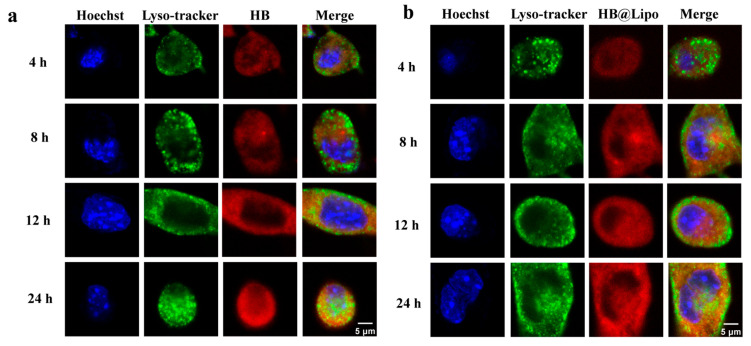

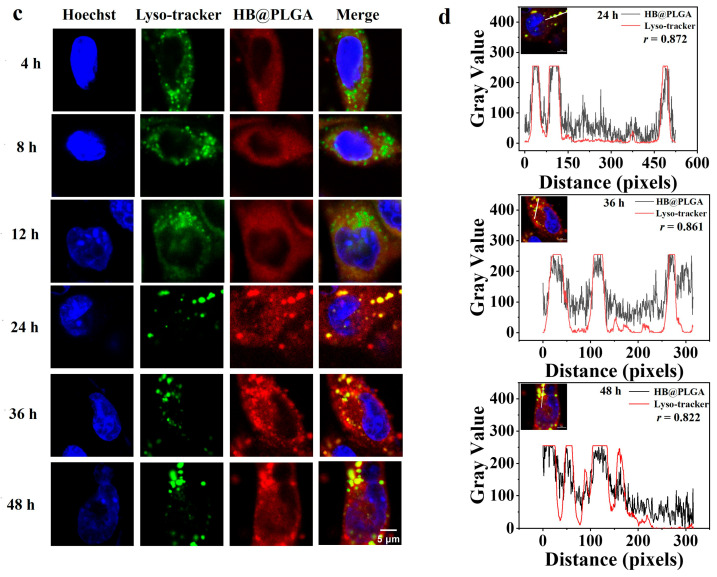
Representative CLSM images demonstrating the distribution of HB (**a**), HB@Lipo (**b**), and HB@PLGA (**c**) in the lysosome of B16 cells. Scale bar: 5 μm. (**d**) The colocalization analysis using ImageJ software. The colocalization in each group was quantified by Pearson correlation coefficient (*r*).

Lysosomes, as the primary catabolic compartments of eukaryotic cells, are responsible for breaking down extracellular substances that have been internalized through endocytosis, or the intracellular components that have been sequestered via autophagy [39]. Presently, the lysosomes of B16 cells were labeled with Lyso-Tracker Green, and the colocalization of free HB, HB@Lipo, and HB@PLGA within lysosomes was observed at 4, 8, 12, and 24 h. The results demonstrated that neither HB nor HB@Lipo exhibited a significant colocalization with lysosomes during 24-h observation period (Figure 6a,b). This suggested that HB and HB@Lipo were not transported to lysosomes during this timeframe. Interestingly, HB@PLGA displayed a distinct behavior after entering cells. By 12 h, no obvious colocalization between HB@PLGA and the lysosomes was observed. However, by 24 h, the green lysosomal fluorescence and red HB fluorescence overlapped significantly, exhibiting a distinct yellow colocalization signal. To further explore this phenomenon, the observation period was extended to 36 h and 48 h. The results indicated that HB@PLGA remained colocalized within lysosomes (Figure 6c). Overly analysis of the red HB fluorescence and the green lysosomal fluorescence was conducted and demonstrated in Figure 6d. The high Pearson correlation coefficient (*r*) strongly demonstrated a consistent and significant colocalization of HB@PLGA within the lysosomes. This phenomenon indicated a different intracellular trafficking pathway of HB@PLGA from that of HB and HB@Lipo. PLGA nanoparticles have been reported to be internalized into cells via endocytosis pathways and preferentially localize within the endosomes of the cells and finally translocated into lysosomes [40]. This previously reported information provided support for the significant colocalization of HB@PLGA with the lysosomes as observed in the current study.

### 3.5. Distribution of HB, HB@Lipo and HB@PLGA in 3D Tumor Spheroids

**Figure 7:**
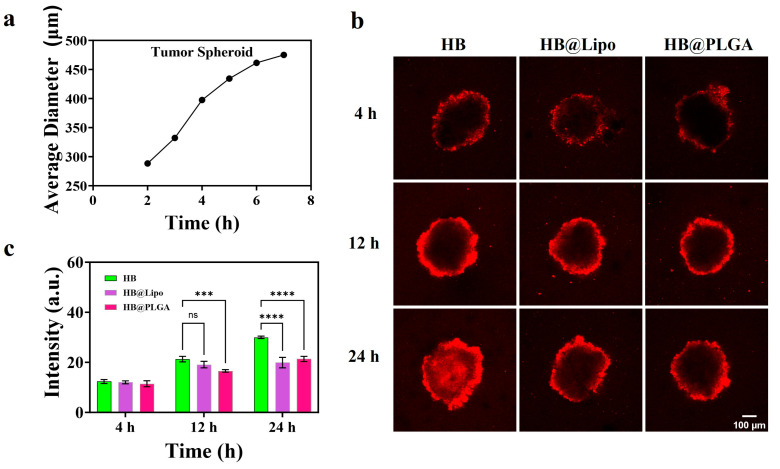
(**a**) Growth curves of tumor spheroids cultured from B16 cells; (**b**) Distribution of HB, HB@Lipo, and HB@PLGA within tumor spheroids observed using CLSM. (**c**) Semi-quantification fluorescence analysis of HB, HB@Lipo, and HB@PLGA in tumor spheroids using ImageJ. B16 tumor spheroids were incubated with HB, HB@Lipo, or HB@PLGA at an equivalent HB concentration of 2 μM for 4, 12, and 24 h. A series of confocal *Z*-stack images were captured from the top to the bottom of the spheroids with the central cross-sectional views displayed. *** *p* < 0.001, **** *p* < 0.0001. Scale bar: 100 μm.

Tumor spheroids, as one of the commonly utilized 3D models, have emerged as a revolutionary and indispensable tool for in vitro biological research. Compared to traditional two-dimensional (2D) tumor cell cultures, the tumor spheroids are capable of more accurately mimicking the physiological environment of tumors, crucial for understanding tumor behavior and developing effective therapeutic strategies [41]. In this study, tumor spheroids were cultured using ultra-low attachment plates. The growth kinetics of the tumor spheroids were carefully monitored. By Day 4, the spheroids reached a major axis length of approximately 400 μm and continued to grow at a rate of 25 μm per day thereafter. This consistent growth pattern suggested the reliability and effectiveness of the 3D culture system (Figure 7a).

Then, tumor spheroids were incubated with HB, HB@Lipo, or HB@PLGA with an equivalent HB concentration of 2 μM and the distribution of HB, HB@Lipo, or HB@PLGA was observed at 4, 12, and 24 h under a confocal microscope based on the red HB fluorescence. A series of confocal *Z*-stack images were acquired from the top to the bottom of the spheroids. Figure 7b displays the central cross-sectional view from each group. The results revealed a difference in the penetration ability among HB, HB@Lipo, or HB@PLGA. Collectively, HB exhibited the highest fluorescence signal intensity within the tumor spheroids, indicating its superior penetration capability compared to HB@Lipo or HB@PLGA. HB@Lipo and HB@PLGA were internalized within tumor spheroids at a significantly lower rate than HB. Figure 7c shows a semi-quantitative analysis of HB or HB nanoformulation uptake by tumor spheroids using image analysis software.

### 3.6. Photodynamic Effects of HB, HB@Lipo and HB@PLGA In Vitro

**Figure 8:**
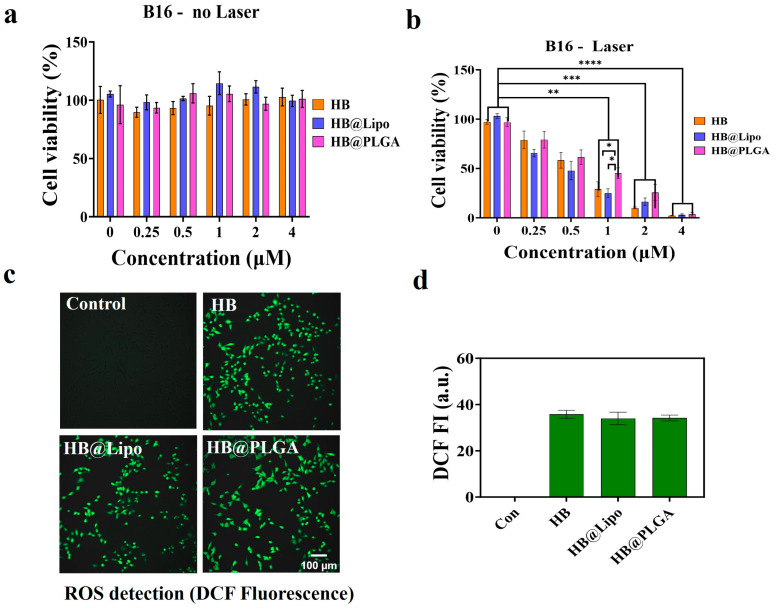
Photodynamic effects of free and encapsulated HB on B16 cells. (**a**) Effect of HB, HB@Lipo, and HB@PLGA on B16 cell viability under the dark condition; (**b**) Cytotoxicity of HB, HB@Lipo, and HB@PLGA on B16 cells upon light illumination; (**c**) ROS detection in the cells treated with free and encapsulated HB upon light illumination; (**d**) Semi-quantitative analysis of DCF fluorescence in each group using ImageJ software. * *p* < 0.1, ** *p* < 0.01, *** *p* < 0.001, **** *p* < 0.0001. Scale bar: 100 μm.

The photodynamic effects of free HB, HB@Lipo, and HB@PLGA on B16 cells were explored. The results from the MTT assay demonstrated that under non-irradiation conditions (Figure 8a), HB, HB@Lipo, and HB@PLGA, all had no obvious cytotoxicity in B16 cells with concentrations up to 4 μM. Next, their dose-dependent effects on B16 cell viability were explored upon laser illumination. B16 cells were exposed to HB, HB@Lipo, or HB@PLGA with concentrations ranging from 0 to 4 μM for 6 h, followed by irradiation for 2 min (0.5 W/cm^2^). Then cells were further cultured until 24 h to allow PDT-induced cytotoxicity to occur. The results demonstrated that upon light exposure, the cell viability of the photosensitizer-exposed cells was obviously decreased, confirming the photodynamic killing of free and encapsulated HB. Increased concentrations of photosensitizers induced more potent cell-killing effects, exhibiting a remarkable dose-dependent effect. The light alone failed to decrease the cell viability. Moreover, at the concentration of 1 μM, slight statistical differences were detected between the free HB group and HB@PLGA group, as well as between the HB@Lipo group and HB@PLGA group, exhibiting a slightly lowered phototoxicity induced by HB@PLGA compared to the free HB and HB@Lipo. However, this difference was abolished when the drug exposure concentrations were elevated to 2 μM or 4 μM, which highlighted the correlation between intracellular HB accumulation and the photodynamic killing effect (Figure 8b).

Meanwhile, intracellular ROS was detected in photosensitizer-exposed cells upon laser irradiation. The cells were incubated HB, HB@Lipo, or HB@PLGA at an equivalent HB concentration of 1 μM for 6 h, followed by 2 min of laser irradiation. Then cells were cultured for an additional 2 h to allow ROS generation. As demonstrated in Figure 8c, no green fluorescence of DCF was detected in the control cells that received irradiation alone. In contrast, upon irradiation, obvious green fluorescence of DCF was detected in the cells that were incubated with photosensitizers, revealing a remarkable intracellular ROS generation. Semi-quantitative analysis of DCF fluorescence in the cells of each group demonstrated the substantial ROS production triggered by free and encapsulated HB after irradiation (Figure 8d).

Collectively, free HB, HB@Lipo, and HB@PLGA all achieved a potent photodynamic cell-killing effect on tumor cells with substantial ROS production.

## 4. Discussion

PDT has been established as an effective therapeutic modality for treating melanoma. In this study, two types of nanocarriers—liposomes and PLGA nanoparticles—were utilized to encapsulate the photosensitizer HB. During nanoparticle preparation, HB-loaded liposomes with controlled particle sizes were prepared via the thin-film hydration method followed by extrusion through polycarbonate membranes. When preparing PLGA nanoparticles, due to the complexity of influencing factors, a single-factor experimental design was adopted. The concentrations of PLGA and PVA in the internal aqueous phase were pinpointed as the most crucial variables. The PVA concentration in the internal aqueous phase exerted a significant impact on the system viscosity as well as the consequent encapsulation efficiency. An appropriate increase in the concentration of PLGA led to a rise in the viscosity of the oil phase. This increased viscosity posed a significant challenge for disintegrating the compound emulsion droplets. As a result, larger nanoparticles were formed. Similarly, a reduced PVA concentration in the internal aqueous phase could decrease the system viscosity but lead to an abrupt decline in encapsulation efficiency. This phenomenon was also attributed to the insufficient difference in PVA concentration between the internal and external aqueous phases, which impaired the ability of the system to effectively encapsulate HB. Therefore, an appropriate combination of PLGA concentration and PVA concentration was expected to balance the encapsulation efficiency and the particle size of the resulting nanoparticles. Some other reports also revealed that PVA was an important formulation parameter for modulating the pharmaceutical properties of PLGA nanoparticles [42,43], consistent with our present findings.

Notably, photodynamic action may proceed through two distinct mechanisms related to oxygen dependencies: Type I reactions generate oxygen-independent radicals (superoxide/hydroxyl) via electron transfer, demonstrating particular efficacy in hypoxic tumor microenvironments. In contrast, Type II reactions strictly require molecular oxygen to produce singlet oxygen through energy transfer and are dominant under normoxic conditions [44]. The results from the determination of the singlet oxygen (^1^O_2_) production induced by HB demonstrated that HB belongs to the Type II photosensitizer at least. Moreover, encapsulation within nanocarriers did not compromise the singlet oxygen (^1^O_2_) production capability of HB. Some research supported our study that HB played the photodynamic action mainly through the type II mechanism by the trigger of singlet oxygen (^1^O_2_). However, this research also pointed out that the newly prepared HB could efficiently produce hydroxyl radicals, with the type I mechanism involved [45].

In cellular uptake studies, cells were incubated with HB, HB@Lipo, and HB@ with an equivalent HB concentration of 2 μM for different time periods. Flow cytometry analysis revealed that both free HB and HB@Lipo exhibited a rapid cellular uptake and achieved their highest uptake amount at 6 h. The cellular uptake of HB@PLGA exhibited a relatively slower and continued cellular uptake until 24 h. Notably, all formulations exhibited comparable overall cellular uptake levels at 24 h. This finding suggested that the nanocarriers might influence the cellular uptake and distribution of drugs. Our observation was also supported by other studies. A study on the cellular uptake of free and liposomal doxorubicin found that cells internalized free doxorubicin manifold faster than liposomal doxorubicin [46]. The divergence in the uptake kinetics can likely be attributed to the different cellular uptake mechanisms. Hydrophobic and lipophilic HB diffuses into the cells. Liposomes are primarily internalized via endocytosis. Besides, some liposomes enter the cells via membrane fusion where the lipid bilayer of the liposomes directly fuses with the phospholipid bilayer of the cell membrane, thus achieving an efficient delivery [47]. However, this pathway is highly dependent on the composition of lipids and the particular triggering conditions. As a prevalent uptake pathway, the endocytosis of liposomes is also size-dependent with clathrin-dependent uptake for the larger liposomes and dynamin-dependent for the relatively smaller liposomes [48,49]. PLGA nanoparticles are reported to be taken up mainly via endocytosis pathways [50]. Compared with the traditional 2D tumor cell culture model, the study on tumor spheroids revealed distinct cellular uptake patterns. The penetration rate of free HB was obviously lowered and showed continuous internalization into the cell within 24 h. This disparity may arise from the differences between 2D monolayers and tumor spheroids. HB can easily penetrate into the single-layer cultured tumor cells while the multi-layered and compact structure of tumor spheroid hinders the drug permeability. Among free HB, HB@Lipo, and HB@PLGA, free HB still had the most rapid uptake and penetration rates. The encapsulated HB, both HB@Lipo and HB@PLGA, showed reduced permeability in tumor spheroids compared to free HB. As discussed above, these different cellular uptake mechanisms influence the rate and the extent of internalization within a given time frame. The differences in the internalization pathways and efficiency of HB, HB@Lipo, and HB@PLGA are likely to be more obviously manifested in tumor spheroids due to the requirement of penetrating the multi-layered and compact structure of tumor spheroids. In the following study, we aim to systematically explore the cellular uptake mechanisms of HB, HB@Lipo, and HB@PLGA to provide a deeper understanding of these processes.

The subcellular localization of HB, HB@Lipo, and HB@PLGA was explored in the current study. Previous research has addressed the subcellular localization of HB and its analog. For instance, HB was reported to exhibit a diffuse or punctate distribution in the cytoplasm of several tumor cell types [51]. HB was reported to be predominantly localized in lysosomes, with minor presence in the endoplasmic reticulum, and almost no detection in the nucleus or mitochondria. In HepG2 cells, HB was shown to be widely distributed across mitochondria, endoplasmic reticulum, and the Golgi apparatus [52]. Similarly in U373 MG cells, the photosensitizer hypericin colocalized significantly within the endoplasmic reticulum and Golgi apparatus [53]. Furthermore, the photosensitizer hypocrellin A was observed to overlap significantly with mitochondria in A549 cells [54]. Nevertheless, none of these reports have mentioned the potential localization of HB in lipid droplets. Maybe the exploration of lipid droplets has been neglected based on the perception that lipid droplets are characteristic of adipocytes rather than tumor cells. Actually, lipid droplets are much more than simple sites for fat storage. They have gained great attention for their crucial roles in cellular metabolism as dynamic organelles in recent years. In the present study, we found that both free and encapsulated HB preferentially accumulated in the lipid droplets of the cells. To date, the interaction between HB and lipid droplets remains uncharacterized. Only research reported early in 1991 revealed that HA (structurally analogous to HB) significantly perturbed the lipid bilayer of the erythrocyte membrane and influenced membrane fluidity. At high concentrations, HA molecules were predominantly located in the mid-region of the membrane lipid bilayer [55]. Given the lipophilic characteristics of both HB and HB@Lipo, along with the structural similarity between liposomes and cell membranes, a similar interaction between HB and lipid substance may occur within lipid droplets. For HB@PLGA, the mechanism underlying its trafficking to lipid droplets also remains unclear. No direct reports have addressed the interaction between PLGA and lipid droplets. Nevertheless, a study on PLGA–lipid nanocomposites indicated that the electrostatic attraction between the two components acted as the major driving force for the formation of polymer–lipid hybrids [56]. Presently, we propose an explanation for HB@PLGA localization within lipid droplets. The PLGA used in our study possesses overall hydrophobic properties, due to the hydrophobic groups (such as the lactate units) in its structure. The hydrophobicity of the PLGA structure enables the encapsulation of hydrophobic HB via hydrophobic interaction. During the preparation of HB@PLGA, the employment of PVA introduces the hydroxyl group [57], endowing the nanocarrier with amphiphilic properties. The utilization of PLGA as the nanocarrier improves biocompatibility. The lipid droplets, ubiquitous in cells, consist of a single layer of phospholipid molecules. The hydrophilic heads of the single-layer phospholipids face outward while the hydrophobic tails warp inward around the neutral lipid core [58]. It is speculated that the amphiphilic properties of HB@PLGA nanoparticles, after entry into the cells, interacted with the surface of lipid droplets via the hydrophobic interaction and hydrophobic interaction. This interaction promoted the colocalization of HB@PLGA within the lipid droplets. The dynamic intracellular trafficking and fusion of lipid droplets facilitated the accumulation of HB@PLGA, as evidenced by the large concentrated fluorescent distribution observed in the confocal studies. Moreover, the accumulation of HB and its nanoformulations in lipid droplets would readily lead to robust lipid peroxidation during photodynamic therapy. However, the fate of HB accumulated in the lipid droplets in the absence of laser illumination needed to be tracked further.

In the lysosomal staining experiment, neither HB nor HB@Lipo showed significant colocalization with lysosomes at 24 h. In contrast, HB@PLGA displayed a pronounced lysosomal colocalization at 24 h, which persisted at 36 and 48 h. The distinct internalization pathways of HB, HB@Lipo, and HB@PLGA might explain the observed disparity in their subcellular localization. Previously, PLGA nanoparticles were reported to be taken up mainly via endocytosis pathways. They preferentially localized within the endosomes of the cells and then translocated to the lysosome later. Moreover, the use of polyvinyl alcohol (PVA) during the preparation of PLGA nanoparticles led to the presence of hydroxyl groups on the surface of these nanoparticles [57]. These hydroxyl groups might interact with the cellular pH environment. Specifically, they enhance the recognition of the nanoparticles by the lysosomes and promote the translocation of the nanoparticles into the lysosomes via lysosomal vesicles [59]. When HB is associated with PLGA in the form of HB@PLGA, it is likely that this association influences its trafficking route within the cell. HB@PLGA is guided to follow the typical pathway of PLGA nanoparticles, ultimately resulting in its accumulation within the lysosomes.

We evaluated the phototoxicity of free HB, HB@Lipo, and HB@PLGA. The results revealed that the phototoxicity of HB formulations was tightly associated with the concentrations of photosensitizers accumulated in the cells. ROS detection corroborated the photodynamic effect of HB. The ROS production after the light activation of photosensitizers will induce oxidative stress-related damage, a universal mechanism underlying PDT effects. Oxidative stress-related damage may manifest in various forms, including mitochondria ROS injury, lipid peroxidation, lysosomal destruction, or endoplasmic reticulum stress, all of which are related to the subcellular localization of photosensitizers. Notably, ROS generated by the activated photosensitizers will also trigger the intrinsic anti-oxidative defense system, typically the Nrf2 signaling pathway, which would inevitably compromise the PDT efficacy. Combined with subcellular distribution analysis, we will further explore the types of oxidative stress-related damage and their correlations with PDT efficiency in further study.

## 5. Conclusions

This study systematically compared the free and encapsulated HB in the aspect of photophysical-chemical properties, cellular uptake, subcellular distribution patterns, and photodynamic killing efficacy. Encapsulation into nanocarriers exerted no obvious influence on the photophysical-chemical properties of HB, including the UV-visible absorbance, fluorescence spectra, singlet oxygen (^1^O_2_) production capacity, and photostability.

In 2D-cultured B16 cells and tumor spheroids, free HB exhibited the fastest internalization, while HB@PLGA showed the slowest uptake kinetics. Notably, all HB formulations exhibited comparable overall cellular uptake levels at 24 h. Subcellular localization analysis revealed significant colocalizations of free HB, HB@Lipo, and HB@PLGA with lipid droplets, but minimal localization within mitochondria or endoplasmic reticulum. Notably, HB@PLGA exhibited a strong lysosomal colocalization, distinct from free HB and HB@Lipo, exhibiting a unique intracellular trafficking pathway for PLGA-encapsulated HB. Upon laser irradiation, both free and encapsulated HB induced pronounced phototoxicity with a significant reduction in cell viability and substantial ROS production, confirming the robust PDT effect of HB. The photodynamic killing effect was highly correlated with the intracellular HB content, highlighting the critical role of cellular uptake and accumulation in determining the therapeutic outcomes. Future research can focus on further tracking the cellular uptake mechanism and the subcellular distribution routes of HB, exploring the correlation between subcellular distribution and PDT efficacy, and developing strategies to enhance PDT performance.

## Figures and Tables

**Table 1:** Key parameters for HB@Lipo preparation.

DPPC	Cholesterol	HB Conc.	Organic Solvent	Organic Solvent Removal	Particle Control
12 mg	3 mg	5 μL of 40 mM HB in DMSO	chloroform	rotary evaporation for 2 h	extruder

**Table 2:** Influence of the concentration of PVA in the inner aqueous phase.

No.	Inner Aqueous Phase	PLGA Conc.	EE, %	Average Diameter, nm	Dispersity (*Đ*)
1	1% PVA	10%	0	455 ± 2.77	0.216
2	2% PVA	10%	0	341 ± 2.68	0.23
3	3% PVA	10%	0	579 ± 1.08	0.176
4	4% PVA	10%	73	281 ± 1.26	0.137
5	5% PVA	10%	89	390 ± 0.99	0.11

**Table 3:** Influence of PLGA concentration.

No.	Inner Aqueous Phase	PLGA Conc.	EE, %	Average Diameter, nm	Dispersity (*Đ*)
1	4% PVA	10%	73	281 ± 1.26	0.137
2	5% PVA	10%	89	390 ± 0.99	0.11
3	4% PVA	15%	92	748 ± 2.11	0.283
4	5% PVA	15%	67	445 ± 1.69	0.143
5	4% PVA	20%	89	669 ± 3.06	0.361
6	5% PVA	20%	91	701 ± 2.71	0.297

**Table 4:** Optimal parameters for HB@PLGA preparation.

HB Conc.	PLGA Conc.	Inner Aqueous Phase	External Aqueous Phase	Organic Solvent Removal	EE, %	Average Diameter, nm	Dispersity (*Đ*)
20 μL of 40 mM HB in DMSO	10%	4% PVA	1% PVA	Rotary evaporative for 1 h	73	281 ± 1.26	0.137

## Data Availability

Data are contained within the article.

## Abbreviations

The following abbreviations are used in this manuscript:

PDT	Photodynamic therapy
HB	Hypocrellin B
ROS	Reactive oxygen species
HPD	Hematoporphyrin derivatives
PLGA	Poly(lactic-*co*-glycolic acid)
DPPC	1,2-dipalmitoyl-sn-glycero-3-phosphocholine
ER	Endoplasmic reticulum
